# Characteristics and outcomes of patients receiving Hospital at Home Services in the South West of Sydney

**DOI:** 10.1186/s12913-020-05941-9

**Published:** 2020-11-26

**Authors:** Anthony Hecimovic, Vesna Matijasevic, Steven A. Frost

**Affiliations:** 1 Rosemeadow Community Health Centre, Sydney, Australia; 2 Primary and Community Health South Western Sydney Local Health District, Sydney, Australia; 3 South Western Sydney Local Health District Centre for Applied Nursing Research, Ingham Institute of Applied Medical Research, Western Sydney University, 1-3 Campbell street Liverpool, Sydney, 2170 Australia; 4 Liverpool Hospital, Sydney, Australia

**Keywords:** Hospital at home, Community nursing, And nurse practitioner

## Abstract

**Background:**

Hospital at home (HaH) provides acute or subacute care in a patient’s home, that normally would require a hospital stay. HaH has consistently been shown to improve patient outcomes and reduce health care costs. The characteristics and outcomes of patients receiving HaH care across the South Western Sydney Local Health District (SWSLHD) has not been well described. This project aimed to describe the characteristics and outcomes of HaH services across the SWSLHD.

**Methods:**

The characteristics of patients referred to HaH between January 2017 and December 2019, the indications for HaH, and representation rates to hospital emergency department (ED) will be presented.

**Results:**

Between January 2017 and December 2019 there was 7118 referrals to the local health district’s (LHD) HaH services, among 6083 patients (3094 females, 51%), median age 56 years (Interquartile range (IQR), 40–69). The majority of indications for HaH were for intravenous venous (IV) medications (78%, *n* = 5552), followed by post-operative drain management (11%, *n* = 789), rehab in the home (RiTH) (5%, *n* = 334), bridging anticoagulant therapy (4%, *n* = 261), and intraperitoneal medications (1%, *n* = 100). The requirement for presentation to an ED for care, while receiving HaH only occurred on 172 (2%) of occasions. The average length of HaH treatment was 7-days (IQR 4–16). Rates of presentation to ED for HaH patients have decreased since 2017, 3.4% (95% CI 2.7–4.2%), 2018 2.1% (95% CI 1.5–2.8%), and 2019 1.8% (95% CI 1.3–2.4%), *p*-value for trend < 0.001.

**Conclusion:**

Hospital at Home is well established, diverse, and safe clinical service to shorten, or avoid hospitalisation, for many patients. Importantly, avoidance of hospitalisation can avoid many risks that are associated with being cared for in the hospital setting.

## Background

Hospital at home (HaH) provides acute or subacute care in a patient’s home, that normally would require a hospital admission [1]. Substituting for hospital care, HaH care can include hospital avoidance, fully substituting for in-hospital care, and early discharge followed by care at home, partial substitution [2, 3]. Importantly, HaH has been shown to improve patients outcomes and reduce health care costs [4–6].

Even though HaH has become more popular, concerns with safety, availability and cost of in-hospital care, many clinicians have expressed concerns with access to newer technologies and resources that deliver urgent, life-saving treatment [1]. And, disease specific review of HaH have not shown consistent benefits [7, 8]. For this reason, ongoing research is needed to assess the local safety and effectiveness of HaH services. In particular HaH services are (locally known as Hospital in the Home) commenced in the South West of Sydney in 2008, and currently receive approximately 200–300 patient referrals each month, across the local health district (LHD). South Western Sydney Local Health District (SWSLHD) delivers hospital services to a population of approximately one million people, through five acute public hospitals, that between them, have approximately 230,000 admissions annually. Locally unplanned presentation to hospital while receiving HaH is considered a key performance outcome.

To date, there hasn’t been any formal description of the characteristics and outcomes of patients receiving HaH care across the SWSLHD. Therefore, this project aims to describe the characteristics of patients receiving HaH care across the LHD, and the outcomes, in particular, the duration and types of treatment, and unplanned hospital representation rates.

## Methods

### Subjects and setting

South Western Sydney Local Health District delivers hospital services to a population of approximately one million people, through five acute public hospitals that between them have approximately 230,000 adults admissions each year. Approximately 200–300 patients are referred to HaH each month.

### Referral to Hospital at Home services

Patients across the SWSLHD are referred to HaH after presenting to an emergency department, or acute in hospitals setting. Patients can also be referred to HaH by a community based General Practitioner by the Tripe I Hub (The Intake, Information and Intervention service for Primary and Community Care).

### Operations of service

The HaH service operates across the SWSLHD. The program is overseen by a Nurse Practitioner who also provides resource to both staff and patients. A clinical Nurse Specialist (Grade 2) works with the Nurse Practitioner as well. The service is a collaborative between four Ambulatory Care Services across the area who also supply much of the medical governance [9].

### Delivery of Hospital in the Home (HaH) services

The care of HaH patients is primarily undertaken by community health centres across the local health district, using a multi-disciplinary team of nurses, allied health and medical staff. Rehab in the home (RiTH), is a recent addition to the services provided by the HaH Team. The rehab delivered is based around post-operative care and ensures patients can leave hospital post-operatively to receive this care at home.

### Outcomes of interest

Due to the nature of HaH services being to avoid in-hospital care, or reduce length of an episode of hospital care, the main key performance indicator is unplanned hospital presentation while receiving HaH (i.e. presentation to ED for clinical deterioration, or need for vascular access, or to receive care not available in the community setting).

### Statistical analysis

The characteristics of HaH admissions are presented using descriptive statistics. Averages of continuous data are presented as median with interquartile range (IQR). Yearly rates of presentation to ED were compared using Poisson regression, and are presented with 95% confidence intervals (CI) [10]. All levels of statistical significance are set at 0.05, and all data management and analysis were performed using the R language for statistical computing [11].

### Ethical considerations

This project was reviewed by the **South Western Sydney Local Health District Human Research Ethics Committee (HREC)** and was determined to meet the requirements of the Australian National Statement on Ethical Conduct in Human Research (2007). Due to the use of routinely collected hospital separation data, the need for individual patient consent was waived (HREC code - ETH 08739). Permission to access the electronic medical record data was obtained from the Head of Department for Primary and Community Health SWSLHD.

## Results

The characteristics of HaH admissions between January 1st 2017 and December 31st 2019 are presented in Table 1. During this period there was approximately 665,000 hospital separations across the SWSLHD, and 7118 referrals to HaH services, among 6083 patients (3094 females, 51%), median age 56 years (IQR, 40–69). The majority of indications for HaH were for IV medications (78%, *n* = 5552), followed by post-operative drain management (11%, *n* = 789), rehab in the home (5%, *n* = 334), bridging anticoagulant therapy (4%, *n* = 261), and intraperitoneal medications (1%, *n* = 100). The average length of HaH treatment was 7-days (IQR 4–16). Presentation to an emergency department for care, while receiving HaH only occurred on 172 (2%) occasions, a median of 2.3% per month (IQR 1.3, 3.1).

**Table 1:** Characteristics of Hospital in the Home Services, January 2017 to December 2019

	Year of referral			Combined
	2017	2018	2019	
Age (yrs), median (IQR)	58 (42, 70)	58 (42, 71)	55 (38, 68)	57 (41, 70)
Females, % (n)	49% (1202)	52% (1146)	51% (1257)	51% (3605)
Indication for HiTH, % (n)				
IV medications	81% (2006)	76% (1671)	76% (1875)	78% (5552)
Post-op drain	10% (243)	12% (257)	12% (289)	11% (789)
Rehab in the home	3% (67)	6% (125)	6% (142)	5% (334)
Bridging therapy	3% (84)	5% (99)	3% (78)	4% (261)
IP medications	2% (43)	1% (23)	1% (34)	1% (100)
Other (NP review etc.)	1% (23)	1% (20)	2% (39)	1% (82)
Length of treatment (days), median (IQR)	7 (4,17)	7 (4,18)	7 (4,15)	7 (4, 16)
Presentation to ED, % (n)	3% (84)	2% (45)	2% (43)	2% (172)

Presentation rate to ED, between January 2017 and December 2019, based on indication for HaH are presented in Table 2. Patients receiving HaH for Rehab had the highest rates of presentation to ED (4.2%), and overall rates of presentation to ED for HaH patients has decreased since 2017, 3.4% (95% CI 2.7–4.2%), 2018 2.1% (95% CI 1.5–2.8%), and 2019 1.8% (95% CI 1.3–2.4%), *p* for trend < 0.001.

**Table 2:** Presentation to emergency department, based on indication for HiTH, January 2017 to December 2019

	Year of referral			Combined
Indication for HiTH, % (n/N)	2017	2018	2019	
IV medications	3.2% (64/2006)	2.0% (33/1671)	1.6% (30/1875)	2.3% (127/5552)
Post-op drain	4.9% (12/243)	2.3% (6/257)	1.4% (4/289)	2.8% (22/789)
Rehab in the home	7.5% (5/67)	3.2% (4/125)	3.5% (5/142)	4.2% (14/334)
Bridging therapy	3.6% (3/84)	2.0% (2/99)	3.9% (3/78)	3.1% (8/261)
IP medications	0.0% (0/43)	0.0% (0/23)	0.0% (0/34)	0.0% (0/100)
Other (NP review etc.)	0.0% (0/23)	0.0% (0/20)	2.6% (1/39)	1.2% (1/82)
All HiTH, rate (95% CI)	3.4% (2.7–4.2)	2.1% (1.5–2.8)	1.8% (1.3–2.4)	*p* for trend < 0.001

## Discussion

This study has been able to describe the characteristics and outcomes of patients receiving HaH services across the SWSLHD between January 2007 and December 2019. The majority of these patients were referred to HaH for administration of IV medications, but also included management of post-operative drains, bridging of anticoagulant therapy (before and after a surgical admission), and rehab in the home following surgery. Only a very small proportion (3%) of these patients had a change in their clinical status that resulted in them needing to present to an ED during HaH.

The benefit of HaH services to avoid hospitalisation has been widely described [1, 2, 6], and has been shown to be a safe, effective and a cost saving alternative to hospitalisation for a wide range of acute problems [3, 5]. In particular the HaH has been shown to be a preferred preference for acute care by patients [6], and as a result both clinical outcomes and patient reported outcomes have been proposed as measures of the quality of care. HaH services have been associated with lower mortality, reduced risk of hospital readmission, and greater patient and carer satisfaction [1]. However, disease specific review of HaH have not shown consistent benefits, especially among patients with some chronic conditions [7, 8].

The results of this study show a similar low-rate of the need for unplanned presentation to the ED during HaH, among studies in the Australian setting and other parts of the world [1, 12]. And the indications for HaH reported in this study were similar to another Australian study [12], with the majority of patients also being referred to HaH for IV therapy. Importantly, this study has shown a downward trend in the need for presentation to the emergency department during HaH, and increasing number of patients being referred to HaH for rehab, following surgery, in the home. At a local level the results of this study have highlighted the increasing need for HaH services and a continued low number of patients with unplanned presentation to ED during HaH care.

An overall strength of this study is the description of a wide range of HaH patients, across a local health district, over many years. In particular, the HaH team provides services to five major hospitals and general practitioners, that supply health services to the one million residents of the south west of Sydney. The potential to miss patient presentations to hospital during HaH, over the study period, was minimised by both active surveillance of patients under HaH care, by the HaH team, and an extensive search of the hospital electronic patient records to identify ED presentations.

The results of this study highlight the diverse indications for HaH services, and their effectiveness to avoid hospitalisation. The implications of the success of HaH services to shorten, or avoid hospital has many implications for patient safety and outcomes. Importantly, HaH services in the Australian setting have been attributed with the avoidance of the need for increased numbers of acute hospital beds [13]. Avoidance of hospitalisation will lower the risk of hospital related adverse events, infections, delirium and nutritional problems. And, given the high risk of these events amongst the more vulnerable of the population, the elderly, HaH services has many potential direct and indirect benefits.

## Conclusion

Hospital at Home Services across the South Western Sydney Local Health District are predominately for administration of IV medications, but include management of post-operative drains, bridging of anticoagulant therapy, and rehab in the home. Importantly, only a very small proportion (3%) of HaH episodes will include the need for patients to present to ED. Hospital in the Home is well established, diverse, and a safe clinical service to shorten, or avoid hospitalisation for many patients. Importantly, avoidance of hospitalisation can avoid many risks that are associated with being cared for in the hospital setting.

## Data Availability

The datasets used and/or analysed during the current study available from the corresponding author on reasonable request.

## Abbreviations

CI: Confidence interval
ED: Emergency department
HaH: Hospital at home
HiTH: Hospital in the home
HREC: Human Research Ethics Committee
IQR: Interquartile range
IV: Intravenous
LHD: Local health district
RiTH: Rehab in the home
SWSLHD: South Western Sydney Local Health District
